# Artificial miRNA mediated resistance in tobacco against *Jatropha leaf curl Gujarat virus* by targeting RNA silencing suppressors

**DOI:** 10.1038/s41598-020-79134-z

**Published:** 2021-01-13

**Authors:** Prashant More, Parinita Agarwal, Abhishek Anand, Neeti Sanan-Mishra, Pradeep K. Agarwal

**Affiliations:** 1 Plant Omics Division, CSIR-Central Salt and Marine Chemicals Research Institute (CSIR-CSMCRI), Council of Scientific and Industrial Research (CSIR), Gijubhai Badheka Marg, 364 002 Bhavnagar, Gujarat India; 2 Academy of Scientific and Innovative Research (AcSIR), Ghaziabad, 201002 India; 3 Plant RNAi Biology Group, International Centre for Genetic Engineering and Biotechnology, Aruna Asaf Ali Marg, New Delhi, 110 067 India

**Keywords:** Biochemistry, Molecular biology, Biotechnology, Plant biotechnology, Physiology, Metabolism, Plant sciences, Biofuels, Photosynthesis, Plant stress responses

## Abstract

The leaf curl disease of Jatropha caused by geminiviruses results in heavy economic losses. In the present study, we report the identification of a new strain of a *Jatropha leaf curl Gujarat virus* (JLCuGV), which encodes six ORFs with each one having RNA silencing suppressor activity. Therefore, three artificial microRNAs (amiRNAs; C1/C4, C2/C3 and V1/V2) were designed employing overlapping regions, each targeting two ORFs of JLCuGV genomic DNA and transformed in tobacco. The C1/C4 and C2/C3 amiRNA transgenics were resistant while V1/V2 amiRNA transgenics were tolerant against JLCuGV. The relative level of amiRNA inversely related to viral load indicating a correlation with disease resistance. The assessment of photosynthetic parameters suggests that the transgenics perform significantly better in response to JLCuGV infiltration as compared to wild type (WT). The metabolite contents were not altered remarkably in amiRNA transgenics, but sugar metabolism and tricarboxylic acid (TCA) cycle showed noticeable changes in WT on virus infiltration. The overall higher methylation and demethylation observed in amiRNA transgenics correlated with decreased JLCuGV accumulation. This study demonstrates that amiRNA transgenics showed enhanced resistance to JLCuGV while efficiently maintaining normalcy in their photosynthesis and metabolic pathways as well as homeostasis in the methylation patterns.

## Introduction

Viruses have emerged as major plant pathogens causing severe losses to agricultural production worldwide^1^. Geminiviridae is the second largest family of plant viruses^2^, possessing the potential to exploit a broad range of plants as its host. The geminiviruses are characterized by geminate virus particles that are categorized into nine genera based on their genome organization, insect vector, host range and phylogeny reconstruction, viz., *Becurtovirus, Begomovirus, Curtovirus, Eragrovirus, Mastrevirus, Topocuvirus, Turncurtovirus, Capulavirus and Grablovirus*. Based on the number of DNA molecules in the viral genome, they can be: monopartite (one DNA molecule) or bipartite (two DNA molecules: DNA-A and DNA-B)^3^. The viral genome in monopartite viruses and the DNA-A component of bipartite viruses are homologous and have a similar organization that includes the V1/AV1, C1/AC1, C2/AC2, C3/AC3, and C4/AC4 ORFs^3^.

The geminiviruses infect a wide range of economically important crops like cassava, cotton, grain legumes and tomato, causing significant financial losses across the world. These economic losses have been estimated to about US$ 1300–2300 million for cassava in Africa^4^, US$ 5 billion for cotton in Pakistan, US$ 300 million for grain legumes in India^5^ and the US$ 140 million in Florida for tomato alone^6^. The DNA viral mutation rates roughly range between 10^–8^ and 10^–6^ substitutions per nucleotide per cell infection^7^. This high rate of recombination and mutation leads to their great diversity worldwide^8^, thereby increasing their host range and geographical distribution. Thus, geminiviruses are emerging as widely spread and diverse plant DNA viruses posing severe threat to crop production.

Jatropha, belonging to family Euphorbiaceae, has various medicinal properties^9^. It has also gained importance as a biodiesel producing crop as Jatropha seeds contain 27–40% oil^10^. The oil yield is affected by attacks of various pathogens such as viruses, bacteria, fungi and nematodes. The leaf curl disease in Jatropha, caused by the whitefly transmitted geminiviruses, is the most devastating. In India, it causes a reduction of up to 80% in seed yield^11^ and 40% in oil production^12^. The symptoms of the disease include mosaic and curling of leaves and malformed fruits. To date, both monopartite and bipartite geminiviruses have been detected in Jatropha from India, Kenya, Nigeria, Dominic Republican, Jamaica and the United States of America (USA)^13^.

For generating resistance to geminiviruses, various molecular strategies based on RNA silencing and gene editing have been reported including antisense RNAs^14^, pathogen-derived resistance^15^, expression of double-stranded (ds) RNA homologues^16^ and CRISPR/Cas9 system^17^. The RNA silencing mechanism in plants forms the first line of defence by targeting the viral mRNA or the RNA genome^18^. Interestingly, viruses have evolved to acquire a variety of unrelated RNA silencing suppressors (RSSs) that affect different yet multiple steps in the silencing pathway^19^ to favour virus replication. Several reports have demonstrated that different geminiviral proteins encode RSS activity^20–22^ and may supplement each other to overcome host RNA silencing response. A molecular strategy to target the virus-encoded suppressors is expected to boost plant tolerance to infection. The artificial miRNA (amiRNA) provide a highly specific alternative to silence the viral transcripts for targeting the RSS for developing disease resistance^23^.

Therefore, in this study, we adopted the strategy of identifying and inactivating the virus-encoded RSSs to boost Jatropha plant resistance against geminiviruses. For this purpose, amiRNAs were designed from genomic regions producing overlapping ORFs to target multiple viral transcripts simultaneously.

## Results

### Characterization of geminivirus infecting *Jatropha curcas*

The geminivirus infected leaves were collected from *J. curcas* CP9 accession (IC 565735), from CSIR-CSMCRI Jatropha plantation fields, Neswad (Gujarat, India). The infected plants showed severe to moderate leaf curling with > 70% disease incidence.

For identification of the virus, the infected leaves were analyzed for the presence of DNA-A, DNA-B and DNA-β. Only DNA-A was amplified, and no amplification was observed for DNA-B and DNA-β. This led us to infer that the viral species infecting Jatropha CP9 accession was probably monopartite. For confirmation, the complete genome was amplified by RCA (rolling circle amplification) and sequenced. Sequence analysis confirmed the presence of only one genomic DNA (KM411359, 2758 bp) in the isolated virus. Based on sequence homology to other begomoviruses, it was identified to be a novel isolate and named as *Jatropha leaf curl Gujarat virus* (JLCuGV). Its genomic DNA possessed two ORFs in the sense strand (AV1 and AV2) and four ORFs in complementary strand (AC1, AC2, AC3 and AC4; see Supplementary Fig. S1).

### JLCuGV genes function as RNA silencing suppressors

**Figure 1:**
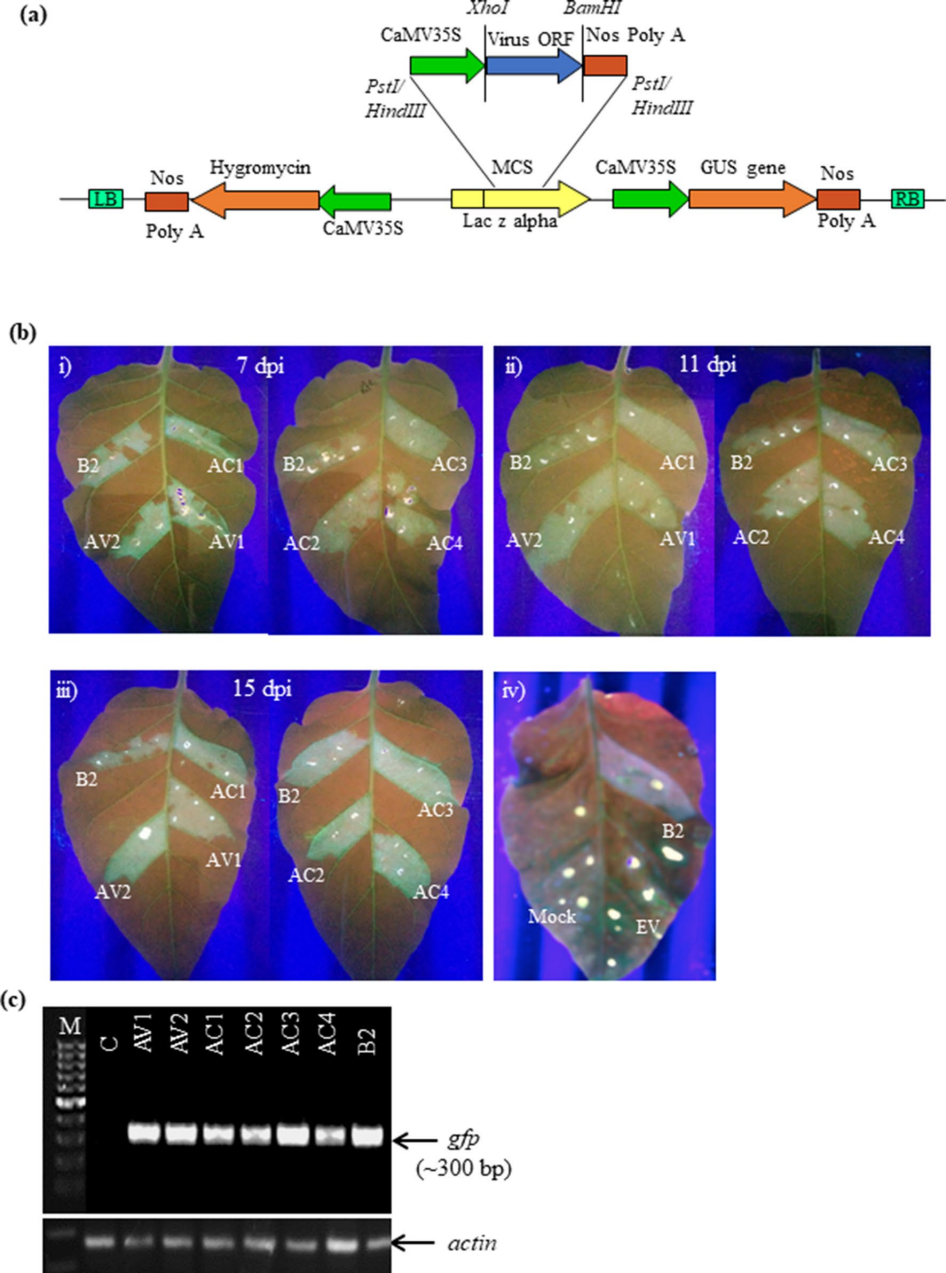
Identification of RNA silencing suppressor activity in the ORFs of JLCuGV genomic DNA. (**a**) The schematic representation to show the cloning strategy of individual JLCuGV ORFs in pCAMBIA-1301. (**b**) Pictures of leaves showing restoration of GFP fluorescence on agroinfiltration of AV1, AV2, AC1, AC2, AC3 and AC4 after (i) 7 dpi (ii) 11 dpi and (iii) 15 dpi. *Flock house virus* B2 (B2) gene was used as a positive control in each case. (iv) control panel showing the leaves infiltrated with *Flock house virus* B2 (B2) gene as a positive control and empty vector (EV) and Mock (only buffer) as a negative control at 15 dpi. (**c**) Amplification of *gfp* gene (upper panel) and *actin* gene (loading control; lower panel) from the cDNA isolated from leaves agroinfiltrated independently with each JLCuGV ORFs (AV1, AV2, AC1, AC2, AC3 and AC4), B2: *Flock house virus* B2 gene (positive control), C: Control mock (only buffer infiltrated leaf).

For identification of potential RSS in the JLCuGV, the six ORFs of the genome (AV1, AV2, AC1, AC2, AC3 and AC4) were individually cloned in pCAMBIA-1301 (Fig. 1a) and were independently agroinfiltrated in the in-house-developed stable green fluorescent protein (GFP)-silenced tobacco lines. The presence of strong green fluorescence indicated a reversal of GFP silencing. The assay was performed using the well-characterized RSS, FHVB2 (*Flock house virus* B2 gene), as a positive control^24^ and empty vector and mock (only buffer) infiltration as a negative control [Fig. 1b(iv)]. The infiltrated leaf patches were observed under UV at regular intervals of 7, 11 and 15 dpi (days post-inoculation; Fig. 1b(i–iii)). The GFP fluorescence was obtained with all six JLCuGV genes indicating that all the ORFs contained inherent RSS activity. This was confirmed by checking for the presence of GFP transcripts in the corresponding infiltrated regions (Fig. 1c). The cDNA generated from the leaves infiltrated with FHVB2 or individual JLCuGV ORFs showed a ~ 300 bp band for GFP transcript, but no band amplified from the mock control. However, detailed molecular analysis is required to characterize the strength, duration and complementation of the RSS activity exhibited by individual ORFs.

### Tobacco transgenic plants expressing amiRNAs display resistance against JLCuGV

#### Confirmation of the transgenics

For developing an effective antiviral strategy, amiRNA constructs were designed to target the RSS. In the genomic region, the ORFs arise from overlapping regions (C1/C4, C2/C3 and V1/V2); so, these regions were selected as targets for the designing of amiRNAs. Three amiRNAs (C1/C4, C2/C3 and V1/V2) were designed in silico (Table 1). The amiRNA constructs were generated by overlapping PCRs and cloned in pCAMBIA-1301 backbone containing the *gus* gene under constitutive promoters (Supplementary Fig. S2). The constructs were transformed in *Nicotiana benthamiana* plants by *Agrobacterium tumefaciens*. The transgenic leaves of T_1_ plants showed complete or partial blue colouration for GUS assay, indicating positive transgenic lines (L61, L64, L67, L73, L79, L95 of C1/C4 amiRNA; L41, L42, L44, L48, L51, L58 of C2/C3 amiRNA; L13, L14, L15, L17, L19 and L21 of V1/V2 amiRNA; Supplementary Fig. S3a). Molecular analysis using gene-specific PCR primers confirmed the presence of *gus* (1.2 kb), *hptII* (1.2 kb) and *amiRNA* (259 bp) genes (Supplementary Fig. S3b). Three putative single-copy transgenic lines of each amiRNA construct (L61, L64 and L67 of C1/C4; L41, L42 and L44 of C2/C3; L13, L14 and L15 of V1/V2 amiRNA) were selected for further analysis (Supplementary Fig. S3c).

**Table 1:** Details of amiRNAs designed against the genes of JLCuGV.

Sr. no	Target genes	Position of overlapping gene sequence (bp)	amiRNA designed (5′–3′)
1	AV1 + AV2	294–472	TCGAAGTTCAGACGGCGACGT
2	AC2 + AC3	1206–1465	TCAGGCGATGAATGGCGTCTA
3	AC1 + AC4	2142–2444	TCAGGGAGCTAAATCCAGCTC

#### Transgenics show resistance against JLCuGV

**Figure 2:**
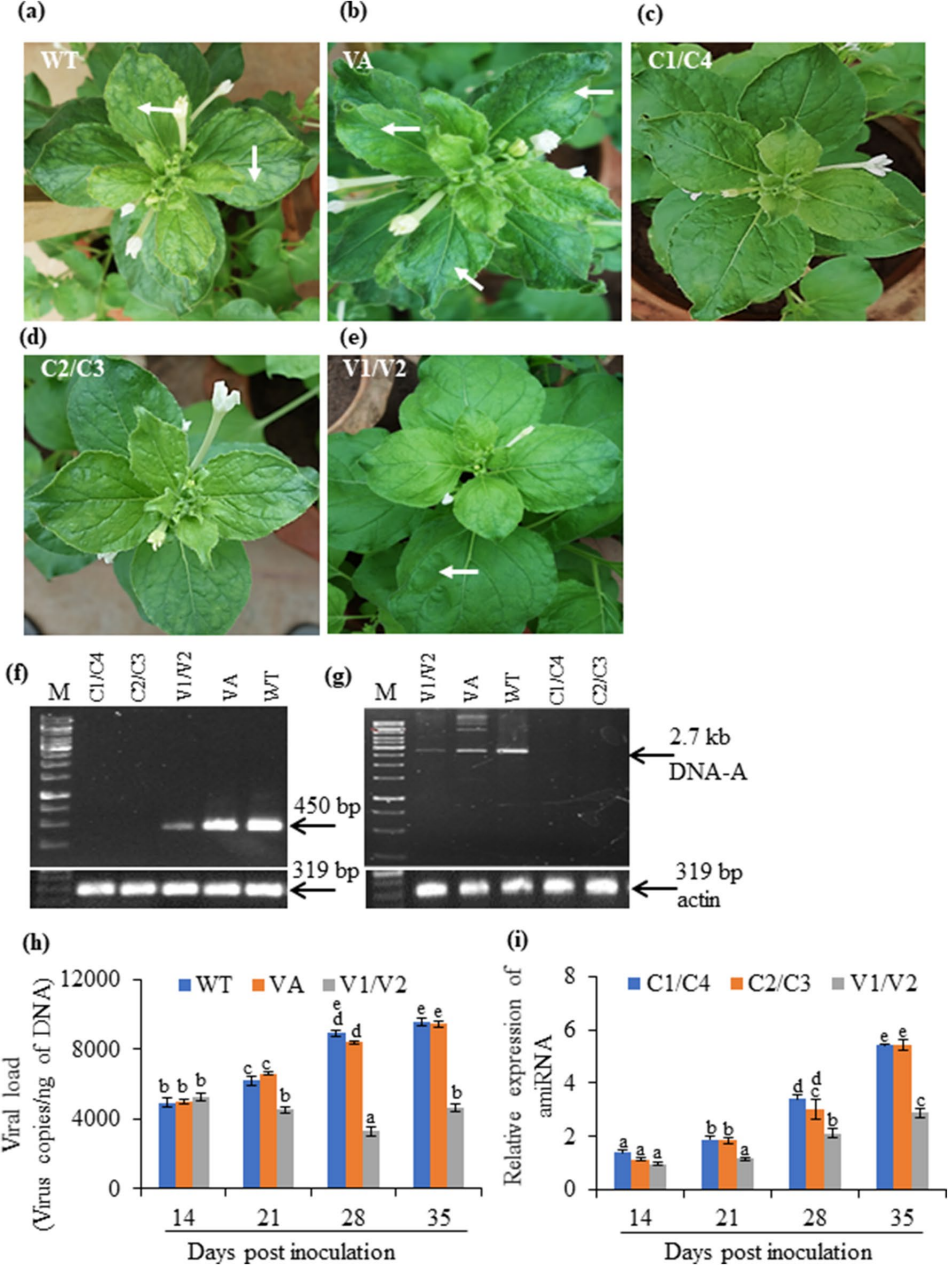
Characterization of the *N. benthamiana* transgenics overexpressing the amiRNA against C1/C4, C2/C3 and V1/V2 ORFs, respectively. Representative pictures to show viral symptoms in (**a**) WT, (**b**) VA, (**c**) C1/C4, (**d**) C2/C3 and (**e**) V1/V2 amiRNA transgenics after 28 dpi. Arrow indicates yellow mosaic/ leaf distortion symptoms. (**f**) PCR amplification of genomic DNA (upper panel) and *actin* gene as a control (lower panel) in agroinfiltrated WT, VA and transgenics for detection of JLCuGV in infected samples (at 28 dpi). (**g**) *Hind*III digested RCA products of the agroinfiltrated C1/C4, C2/C3, V1/V2 transgenics, WT and VA for detection of JLCuGV in infected samples (at 28 dpi). (**h**) The qRT-PCR based estimation of viral load in the infiltrated plants of WT, VA and V1/V2 transgenics after 14, 21, 28 and 35 dpi. (**i**) Relative expression of amiRNA in transgenics. Values are represented as means ± SD (n = 3) and marked with different alphabets (from ‘**a**’ to ‘**e**’) to indicate a significant difference as determined by Tukey test at *P* ≤ 0.05 probability.

To analyze the tobacco plants producing amiRNAs for resistance to virus infection, the agroinfectious clone of JLCuGV (Supplementary Fig. S4) was used for infiltration. The agroinfiltration in WT and VA (vector alone) plants served as control. All the WT (total 3 out of 3 inoculated plants) and VA (total 3 out of 3 inoculated plants) plants showed curl-mosaic symptoms after 21 dpi, and the symptoms increased with an increase in time (Fig. 2a,b). Visible symptoms were not observed in the C1/C4 (L61, L64, L67) and C2/C3 (L41, L42, L44) amiRNA transgenic plants (9 out of 9 plants of each C1/C4 and C2/C3 transgenics; Fig. 2c,d), but 3 out of 9 plants expressing amiRNA V1/V2 (L13, L14, L15) showed weak symptoms on leaves (Fig. 2e).

At 28 dpi, the genomic DNAs from all these plants were checked for the presence of viral DNA by PCR (450 bp) using specific primers (Fig. 2f) and reconfirmed by checking for genomic DNA amplicon (2.7 kb) obtained by *Hind*III digestion of RCA product (Fig. 2g). The *actin* gene was amplified as loading control (Fig. 2f,g). The DNA bands were observed in virus-infected WT and VA plants, but no amplification was seen in case of C2/C3 and C1/C4 amiRNA transgenics. In virus-infected V1/V2 amiRNA transgenic plants, a faint band was obtained.

The WT, VA and V1/V2 amiRNA transgenics, which were found positive for JLCuGV infection, were further checked for the viral load using qRT-PCR for the quantitative determination of infection severity. The viral load of WT and VA was 4959 and 4999 copies/ng, respectively at 14 dpi, which increased by 1.93- and 1.89-fold respectively at 35 dpi (Fig. 2h). However, the viral load of V1/V2 amiRNA transgenics (5364 copies/ng at 14 dpi) decreased by 0.88-fold at 35 dpi (Fig. 2h).

For determining the correlation between the expressed amiRNA levels in transgenics with the JLCGV infection, the quantitative estimation of amiRNA was performed in transgenics using stem-loop qRT-PCR. The level of amiRNA increased from 1.3-fold (C1/C4), 1.15-fold (C2/C3) and 0.97-fold (V1/V2) at 14 dpi to 5.4-fold (C1/C4), 5.4-fold (C2/C3), 2.88-fold (V1/V2) at 35 dpi in amiRNA transgenics (Fig. 2i).

### Methylation profiling of transgenics expressing amiRNA

The genomic DNA from T_1_ amiRNA transgenics of C1/C4 (L61, L67), C2/C3 (L42, L43), V1/V2 (L13, L15), VA and WT plants were used for MSAP (methylation-sensitive amplified polymorphism) analysis with 20 primers, which were earlier characterized to be best polymorphic MSAP primers in tobacco (More et al.^25^). Among 20 primers, 15 primers (Supplementary Table S1) showed consistent and clear profiles. Out of 2520 bands in total, 1528 bands showed methylation. The global methylation events were higher in transgenics as compared to WT and VA. The C1/C4 amiRNA transgenic exhibited very high methylation polymorphism (L67 = 78.73%, L61 = 73.97%), full methylation (L67 = 56.83%, L61 = 40%) and hemi-methylation (L61 = 33.97%, L67 = 21.90%). C2/C3 amiRNA transgenics also showed > 60% methylation polymorphism (L43 = 67.94%, L42 = 61.90%), > 40% full methylation (L42 = 40.63%, L43 = 40.63%) and > 20% hemi-methylation (L43 = 27.30%, L42 = 21.27%). The V1/V2 amiRNA transgenics showed lower values than C1/C4 and C2/C3 amiRNA transgenics for methylation polymorphism (L13 = 54.60%, L15 = 52.06%), full methylation (L15 = 37.46%, L13 = 36.83%) and hemi-methylation (L13 = 17.78%, L15 = 14.60%). The lowest values for methylation patterns (full methylation: 36% and hemi-methylation: 9%), were observed in WT (Table 2).

**Table 2:** The methylation patterns observed in amiRNA transgenics after virus infection.

Type of band	WT	VA	C1/C4 (L61)	C1/C4(67)	C2/C3 (L42)	C2/C3 (L43)	V1/V2 (L13)	V1/V2 (L15)
I	170	158	82	67	120	101	143	151
II	29	36	107	69	67	86	56	46
III	49	63	42	54	48	40	57	67
IV	67	58	84	125	80	88	59	51
TAB	315	315	315	315	315	315	315	315
TMB	**145**	157	233	*248*	195	214	172	164
FMB	116	121	126	*179*	128	128	**116**	118
MSAP (%)	**46.03**	49.84	73.97	*78.73*	61.90	67.94	54.60	52.06
FM (%)	**36.83**	38.41	40.00	*56.83*	40.63	40.63	36.83	37.46
HM (%)	**9.21**	11.43	*33.97*	21.90	21.27	27.30	17.78	14.60

VA and WT were used as controls. The highest values indicated by italic and lowest values indicated by bold.

Type I: Absence of methylation due to the presence of bands in both *Eco*RI/ *Hpa*II and *Eco*RI/ *Msp*I digest;

Type II: Bands present only in *Eco*RI/ *Msp*I digestion but not in the *Eco*RI */ Hpa*II digest;

Type III: Bands present in *Eco*RI/ *Hpa*II digest but not in the *Eco*RI/ *Msp*I digest;

Type IV: Absence of band in both enzyme combinations.

*TAB* total amplified bands, *TMB* total methylated bands, *FMB* fully methylated bands, *FM* Full methylation, *HM* hemi-methylation; the values of TAB = [I + II + III + IV], MB = [II + III + IV], FMB = [II + IV], MSAP% = [(MB/TAB) × 100], FM% = [(FMB/TAB) × 100], HM% = [III/TAB × 100].

For analyzing the changes in methylation patterns of transgenics in comparison to WT, the total bands were further classified as “A to P” (16 combinations; Table 3) based on their banding pattern. The banding pattern was classified into three types: no change (A to D), methylation (E to J) and demethylation (K to P; Table 3). The pairwise comparison between transgenics and WT revealed maximum “no change” and minimum “no change” in VA (65.03%) and C1/C4 amiRNA transgenics L67 (28.76%), respectively (Table 3). The L43 of C2/C3 amiRNA transgenics showed maximum change in methylation (14.51%) pattern compared to WT, whereas the lowest change in methylation pattern (9.59%) was observed in VA. The demethylation percentage was found to be maximum in L67 (45.08%) of C1/C4 amiRNA transgenics, while the lowest demethylation in VA (13.47%) in comparison to WT (Table 3).

**Table 3:** The pairwise comparison of methylation and demethylation pattern of VA, C1/C4, C2/C3 and V1/V2 amiRNA transgenics with WT.

Type of band	Banding pattern				VA	C1/C4 (L61)	C1/C4 (L67)	C2/C3 (L42)	C2/C3 (L43)	V1/V2 (L13)	V1/V2 (L15)
	Transgenic		WT								
	*Hpa*II	*Msp*I	*Hpa*II	*Msp*I							
A	1	0	1	0	22	19	16	20	19	18	21
B	0	1	0	1	40	20	12	33	17	42	38
C	1	1	1	1	139	69	48	103	82	126	128
D	0	0	0	0	50	35	35	45	35	43	37
Total bands					251	143	111	201	153	229	224
**No change (%)**					*65.03*	37.05	**28.76**	52.07	39.64	59.33	58.03
E	1	0	1	1	3	4	3	4	3	7	6
F	0	1	1	1	10	6	8	11	6	4	10
G	0	0	1	1	10	5	10	9	11	9	13
H	0	1	1	0	2	8	7	5	10	0	4
I	0	0	1	0	2	17	7	10	12	9	7
J	0	0	0	1	10	15	20	8	14	11	15
Total bands					37	55	55	47	56	40	55
**Methylation (%)**					**9.59**	14.25	14.25	12.18	*14.51*	10.36	14.25
K	1	1	1	0	13	71	49	41	59	32	20
L	1	1	0	1	20	12	25	11	9	11	17
M	1	1	0	0	4	24	54	21	26	7	11
N	1	0	0	1	2	2	2	1	1	1	3
O	1	0	0	0	3	5	9	5	7	4	0
P	0	1	0	0	10	28	35	13	29	16	10
Total bands					52	142	174	92	131	71	61
**Demethylation (%)**					**13.47**	36.79	*45.08*	23.83	33.94	18.39	15.80

The highest values indicated by italic and lowest values indicated by bold.

### Improved photosynthesis parameters in amiRNA expressing transgenics on JLCuGV infiltration

For understanding the effect of virus infection on the photosynthesis process, the WT, VA and amiRNA transgenics (C1/C4, C2/C3 and V1/V2) were infected (in 3 replicates) with JLCuGV and analyzed for various photosynthetic parameters.

#### Gas exchange related photosynthetic parameters

**Figure 3:**
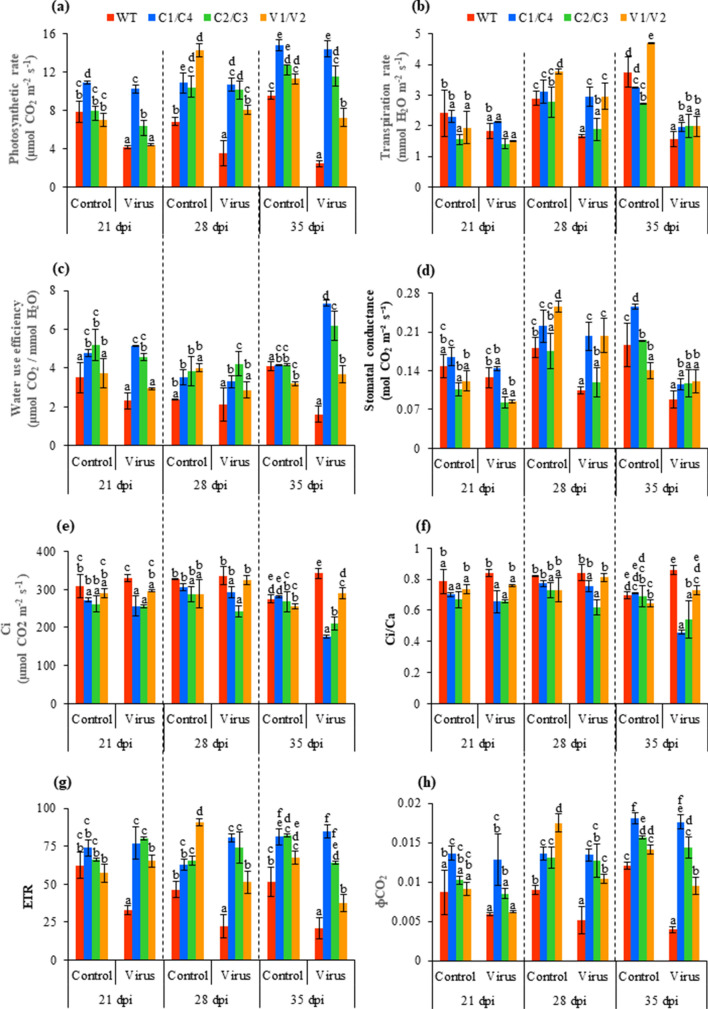
Analysis of gas exchange related photosynthesis parameters of WT, C1/C4, C2/C3 and V1/V2 amiRNA plants with and without JLCuGV agroinfiltration (**a**) Photosynthesis rate, (**b**) Transpiration rate, (**c**) Water use efficiency, (**d**) Stomatal conductance, (**e**) Ci, (**f**) Ci/Ca ratio, (**g**) ETR and (**h**) ΦCO_2_. Values are represented as means ± SD (n = 3) and marked with different alphabets to indicate a significant difference as determined by Tukey test at *P* ≤ 0.05 probability.

The photosynthesis rate decreased significantly on JLCuGV infection in WT and V1/V2 transgenics, while no significant change was observed in C1/C4 and C2/C3 transgenics. In WT, the photosynthesis rate showed a significant decrease of 2.2- and 3.9-fold after 28 dpi and 35 dpi, respectively, as compared to control plants. The virus infiltrated transgenics showed a significantly better photosynthesis rate than the WT at all time points. Among the transgenics, the agroinfiltrated V1/V2 transgenics showed 1.34- and 1.55-fold decrease in photosynthesis rate after 28 dpi and 35 dpi, respectively, as compared to the control plant (Fig. 3a). In C1/C4, C2/C3 and V1/V2 transgenics, the transpiration rate increased significantly by 1.76-, 1.12- and 1.76-fold, respectively, at 28 dpi. The water use efficiency (WUE) and stomatal conductance increased significantly by 4.53-fold (C1/C4 transgenics), 3.11-fold (C2/C3 transgenics) and 2.28-fold (V1/V2 transgenics) by 35 dpi and 1.92-fold (C1/C4 transgenics), 1.12-fold (C2/C3 transgenics) and 1.93-fold (C1/C4 transgenics) at 28 dpi, respectively, as compared to WT (Fig. 3b–d). The internal CO_2_ (Ci) and the ratio of intercellular to ambient CO_2_ (Ci/Ca) increased in virus infiltrated WT and V1/V2 plants, but decreased in C2/C3 and C1/C4 transgenics, as compared to their respective uninfected control plants (Fig. 3e,f). At 35 dpi, the virus infiltrated WT plants showed increased Ci and Ci/Ca values as compared to C1/C4 (1.93- and 1.87-fold, respectively), C2/C3 (1.46- and 1.56-fold, respectively), V1/V2 (1.17- and 1.17-fold, respectively).

On the contrary, the electron transport rate (ETR) and CO_2_ assimilation (ɸCO_2_) decreased in WT and V1/V2 transgenics and increased in C1/C4 and C2/C3 transgenics after virus infiltration. Upon virus infiltration (35 dpi), ETR and ɸCO_2_ increased significantly in C1/C4 (4.12- and 4.47-fold, respectively), C2/C3 (3.46- and 3.63-fold, respectively) and V1/V2 transgenics (1.82- and 2.39-fold, respectively) than WT (Fig. 3g,h).

#### Chlorophyll fluorescence related photosynthetic parameters

**Figure 4:**
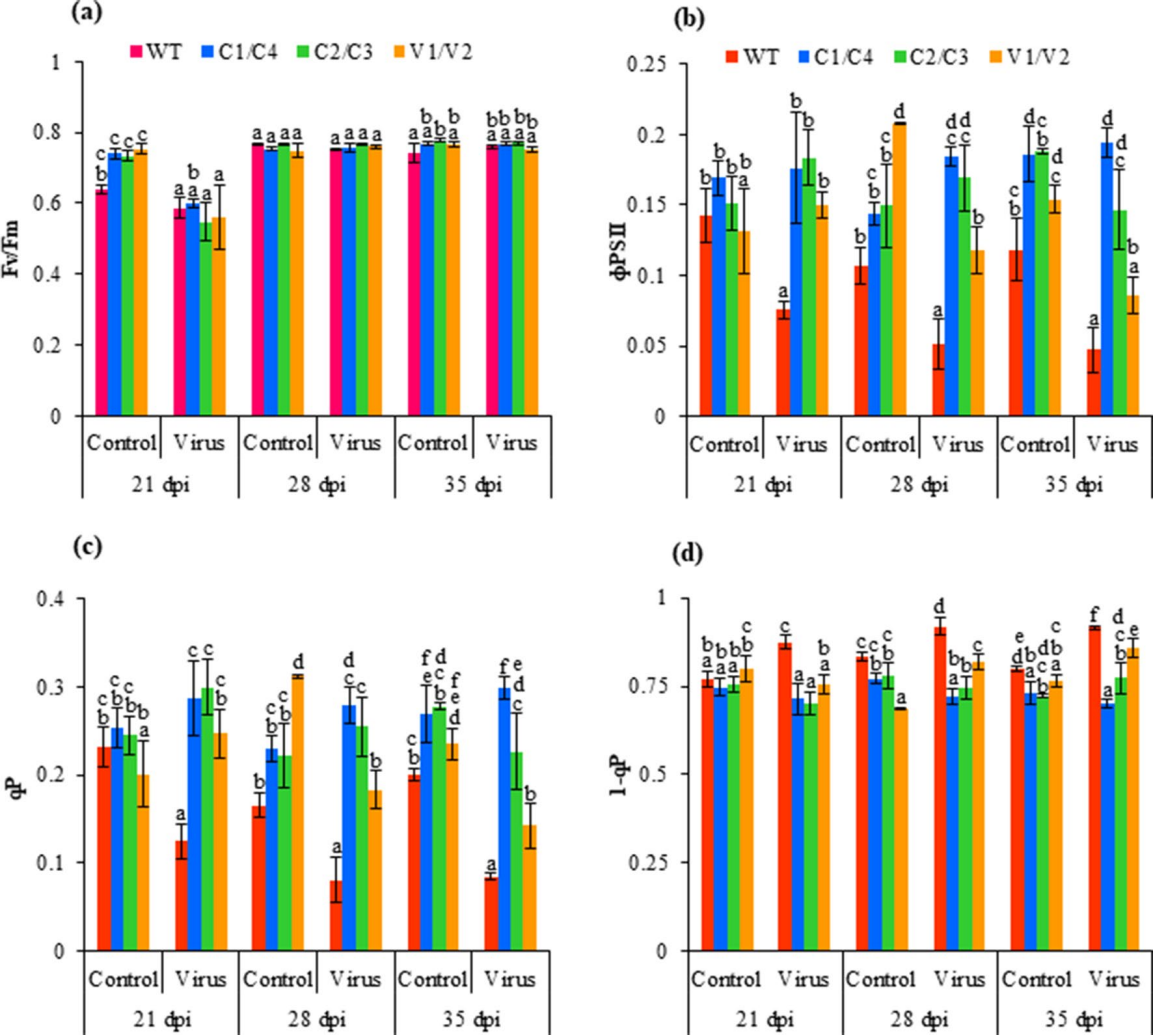
Analysis of chlorophyll fluorescence parameters of WT, C1/C4, C2/C3 and V1/V2 amiRNA plants with and without JLCuGV agroinfiltration. (**a**) ΦPSII, (**b**) Fv/Fm ratio (**c**) qP and (**d**) 1-qP. Values are represented as means ± SD (n = 3) and marked with different alphabets to indicate a significant difference as determined by Tukey test at *P* ≤ 0.05 probability.

The efficiency of photosystem II (Fv/Fm) was similar in both WT and transgenics with and without virus infiltration (Fig. 4a). The quantum yield of PSII electron transport (ɸPSII) and photochemical quenching (qP) increased significantly, while 1-qP significantly decreased in C1/C4, C2/C3 and V1/V2 amiRNA transgenics than WT after virus infiltration at all time points. ɸPSII increased in virus infiltrated C1/C4, C2/C3 and V1/V2 transgenics by 4.12-, 3.11- and 1.82-fold, respectively than WT at 35 dpi (Fig. 4b). Similarly, at 35 dpi, qP also increased on virus infiltration in C1/C4, C2/C3 and V1/V2 amiRNA transgenics by 4.65-, 3.53- and 2.22-fold, respectively, as compared to WT (Fig. 4c). The comparison of the virus infiltrated plant with its control plant showed that ɸPSII and qP decreased in WT and V1/V2 transgenics on virus infiltration, whereas it increased in C1/C4 and C2/C3 amiRNA transgenics. The 1-qP decreased significantly in virus infiltrated C1/C4, C2/C3 and V1/V2 amiRNA transgenics by 1.33-, 1.21- and 1.09-fold, respectively, as compared to WT at 35 dpi (Fig. 4d).

### Metabolite profiling of amiRNA expressing transgenics

**Figure 5:**
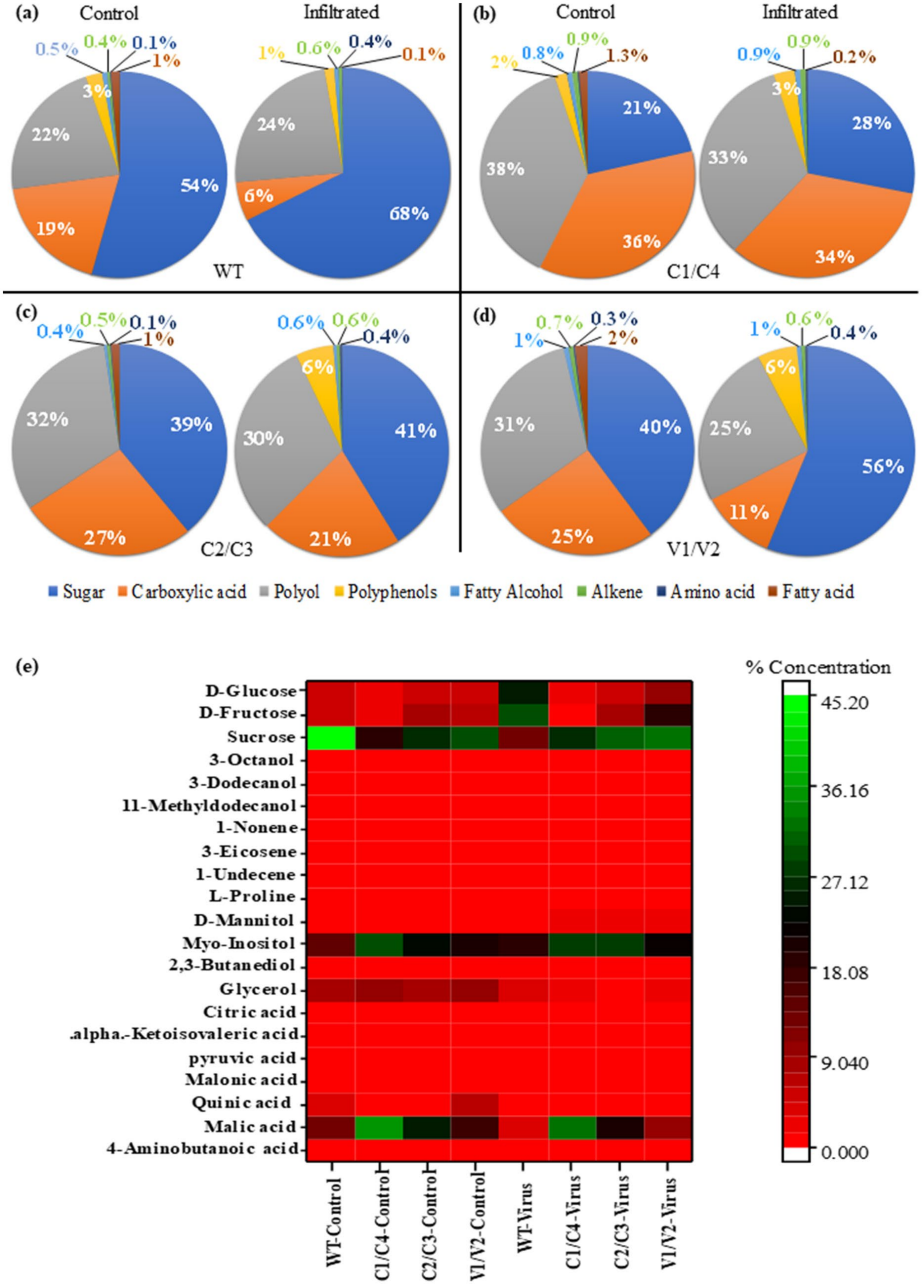
Comparison of different metabolite responses in (**a**) WT, (**b**) C1/C4, (**c**) C2/C3 and (**d**) V1/V2 amiRNA transgenics with and without (control) JLCuGV agroinfiltration, (**e**) heat map of metabolomics with row represents the metabolite and column represents the sample. The metabolite concentrations are shown in different colours.

Metabolite analysis was performed in both WT and amiRNA transgenics with and without JLCuGV agroinfiltration. Twenty-one common metabolites were detected in both healthy and virus infiltrated tissue (Supplementary Table S2). The identified metabolites were categorized into eight different groups like alkenes, amino acids, carboxylic acids, fatty acids, fatty alcohols, polyols, polyphenols and sugars. Sugars formed the largest group, followed by carboxylic acids and polyols. In WT, the concentration of sugar was more on geminivirus infiltration (68%) than the healthy tissue (54%), while the carboxylic acids were more in healthy (19%) than infected tissue (6%; Fig. 5a). In C1/C4 transgenics, healthy and virus infiltrated tissue showed less change in concentration of sugars (21% and 28%, respectively), carboxylic acids (36% and 34%, respectively) and polyols (38% and 33%, respectively; Fig. 5b). Also, in C2/C3 transgenics, healthy and virus infiltrated tissue showed less change in concentration of sugars (39% and 41%, respectively), carboxylic acids (27% and 21%, respectively) and polyols (32% and 30%, respectively; Fig. 5c). While in V1/V2 transgenics, the concentration of sugar was more on geminivirus infiltration (56%) than the healthy tissue (40%), while the carboxylic acids were higher in healthy (25%) than infected tissue (11%; Fig. 5d). The other compounds like polyphenols, fatty acids, fatty alcohols, alkenes and amino acids were less than 6% in both WT and transgenics with healthy and infected tissue.

In amiRNA transgenics, except V1/V2 transgenics, no significant changes were observed in the concentration of metabolites in healthy (control) and virus infiltrated tissues. In WT, C2/C3 and V1/V2 transgenics, fructose and glucose content increased, with a maximum increase of 8.76- and 8.10-fold, respectively, in WT on infiltration. The C1/C4, however, showed a decrease in fructose and glucose content on virus infiltration. The sucrose content increased in transgenics (1.19–1.26-fold), whereas decreased by 1.95-fold in WT with virus infiltration (Table 4). The malic and quinic acid content increased in C1/C4 and C2/C3 transgenics, whereas decreased in WT and V1/V2 transgenics on virus infiltration. The quinic acid content showed a noticeable increase of 3.15-fold and a decrease of 4.29-fold in C2/C3 and V1/V2 transgenics, respectively. The pyruvic acid showed a reduction in both WT and transgenics, with a remarkable decrease of 17.7-fold in WT on virus infiltration (Table 4). In WT, C2/C3 and V1/V2 transgenics, an increase in myo-inositol content (1.23–2.22-fold) was observed. The glycerol content decreased in both WT and transgenics, with a maximum decrease of 4.77-fold in C1/C4 transgenics on virus infiltration (Table 4).

**Table 4:** The fold change in the metabolites in WT and amiRNA transgenics after JLCuGV agroinfiltration.

Sr. no	Class	Compound name	Fold change			
			WT	C1/C2	C2/C3	V1/V2
1	Sugars	Sucrose	− 1.95	+ 1.19	+ 1.26	+ 1.29
2		d-Fructose	+ 8.76	− 1.62	+ 1.13	+ 2.93
3		d-Glucose	+ 8.10	− 1.51	+ 1.06	+ 2.42
4	Carboxylic acids	Pyruvic acid	− 17.70	− 1.75	− 2.04	− 2.97
5		Malic acid	− 1.75	− 1.30	− 1.12	− 1.57
6		Quinic acid	− 1.87	− 1.54	+ 3.15	− 4.29
7		Malonic acid	− 1.42	+ 1.39	− 1.48	− 3.15
8		4-Aminobutanoic acid	− 1.04	+ 13.40	− 1.88	+ 1.75
9		alpha.-Ketoisovaleric acid	− 2.20	+ 2.91	+ 1.16	− 2.73
10		Citric acid	+ 2.06	− 1.18	+ 2.66	+ 6.08
11	Polyols	Myo-Inositol	+ 2.22	− 1.23	+ 1.31	+ 1.23
12		Glycerol	− 1.10	− 3.87	− 4.77	− 3.85
13		d-Mannitol	+ 5.25	+ 3.82	+ 15.35	+ 2.48
14		2,3-Butanediol	+ 1.79	1.21	+ 1.69	1.87
15	Fatty alcohols	11-Methyldodecanol	− 1.09	+ 1.16	+ 1.31	+ 1.53
16		3-Dodecanol	+ 4.64	− 1.35	+ 1.86	+ 1.80
17		3-Octanol	− 1.08	− 1.04	− 1.28	− 1.80
18	Amino acids	l-Proline	+ 1.31	− 1.14	+ 2.43	+ 1.58
19	Alkenes	1-Undecene	+ 1.04	− 1.57	− 2.20	− 2.13
20		3-Eicosene	+ 3.17	− 1.18	+ 3.87	+ 3.36
21		1-Nonene	+ 1.63	+ 2.40	+ 1.58	+ 1.09

The increase and decrease in fold change is indicated by + and − sign, respectively.

Out of all the detected metabolites in the healthy tissue of WT, sucrose (45.09%) was detected in the highest amount, followed by myo-inositol (14.30%) and malic acid (13.52%), while in C1/C4 and C2/C3 transgenics, malic acid (35.06% and 25.17%, respectively) was detected in the highest amount, followed by myo-inositol (29.29% and 23.55%, respectively; Fig. 5e). In virus infiltrated WT tissue, fructose was detected in highest amount (29.85%), followed by glucose (25.30%) and myo-inositol (18.45%; Fig. 5e, Supplementary Table S2), while in virus infiltrated C1/C4 transgenics, malic acid was detected in highest amount (32.34%), followed by myo-inositol (28.55%) and sucrose (26.27%; Fig. 5e, Supplementary Table S2).

## Discussion

This study shows that each of the 6 ORFs of the newly isolated JLCuGV genomic DNA (AC1, AC2, AC3, AC4, AV1 and AV2), independently show strong RSS activity. The reversal of GFP expression in GFP silenced tobacco lines on agroinfiltration of AC1, AC2, AC3, AC4, AV1 and AV2 ORFs, independently, confirmed the strong RSS activity in all the six ORFs. The positive amplification of GFP gene from the cDNA generated from infiltrated leaves, gave the molecular confirmation of RSS activity exhibited by JLCuGV ORFs. The RSS activity acquired by individual JLCuGV ORFs seems to have increased its virulence. In the case of geminiviruses, ORFs like AV2, AC1, AC2, and AC4 were reported as RSSs^26,27^. In *Bhendi yellow vein mosaic virus*, strong RSS activity detected in AC4 and βC1, while low RSS activity is detected for AC2^28^. In the *Tomato leaf curl New Delhi virus*, the RSS activity was identified in AC2, AV2 and AC4 proteins^22^. It was shown that the RSS activity of AC4 and AV2 appeared during early infection and supplemented the RSS activity of AC2 to sustain the response for a longer duration^22^. Here, we report for the first time that all six ORFs of JLCuGV genomic DNA have acquired a strong RSS activity, although detailed molecular analysis is required to characterize the strength, duration and complementation of the RSS activity exhibited by individual ORFs.

As a strategy to boost plant resistance to virus infection, we designed amiRNAs targeting overlapping regions between ORFs C1/C4, C2/C3 and V1/V2 to silence their transcripts and hence inhibit their RSS activity. The *N. benthamiana* transgenics independently expressing C1/C4 and C2/C3 amiRNAs showed resistance against JLCuGV while transgenics expressing V1/V2 amiRNAs showed tolerance to JLCuGV. The WT and VA plants showed high viral load with severe disease symptoms, whereas no viral load and no symptoms were detected in C1/C4 and C2/C3 transgenics. This indicated that JLCuGV resistance in C1/C4 and C2/C3 transgenics could be due to the expression of amiRNA. Several other reports are available that have shown that amiRNAs are effective in bringing out the degradation or silencing of the target viral genes^23,29^.

Interestingly, amiRNA levels were lower in V1/V2 amiRNA transgenics as compared to the C1/C4 and C2/C3 transgenics. The reason needs to be investigated though, in all probability, it may be due to the allelic states of amiRNA precursor, based on the integration of T-DNA into the plant genome, which may have altered the level of amiRNA expression^29^. The lower amiRNA expression level in V1/V2 amiRNA transgenics correlates with the development of mild symptoms and limited tolerance to virus infection. It was observed that the viral load in V1/V2 amiRNA transgenics was much lower than that observed in WT or VA plants. This shows the important role of miRNAs in providing protection from virus infection and the manifestation of pathogen symptoms^30^. Earlier, Vu et al*.*^29^ have shown an inverse correlation between the expression levels of amiRNA and the development of disease symptoms.

DNA methylation plays a major role in protecting plants under both biotic and abiotic stresses. Several reports state that plants can methylate the invading virus genome to suppress their growth^30^. The changes in methylation in *Nicotiana tabacum* genome were observed after the *Cucumber mosaic virus* (CMV) infection^31^^.^ The predominance of CHH hypomethylation has emerged as a major defence response against viral infection^31^. The transgenics C1/C4 and C2/C3 amiRNA exhibited higher methylation across the genome after virus infection as compared to WT, VA and V1/V2 amiRNA transgenics. The higher methylation and demethylation in amiRNA transgenics, corelated with decreased JLCuGV accumulation. This suggested that the homeostasis in methylation pattern in C1/C4 and C2/C3 amiRNA transgenics helped to block the JLCuGV accumulation, leading to resistance against JLCuGV. It has been reported that geminiviruses counteract plant defence strategy by inhibiting DNA methylation. The geminiviral AC2 genes act as RSS by interacting and inhibiting adenosine kinase (ADK), an essential gene of the methylation pathway^32^. ADK plays a role in the production of the methyl group donor S-adenosyl methionine (SAM) and defects in ADK lead to a loss in methylation in plants^33^. As C2/C3 amiRNA transgenics target the AC2 gene, the viral counter defence to suppress ADK was not functional, and the plants could successfully follow the methylation pathway to inhibit the virus growth.

Virus infection usually affects the process of photosynthesis^34,35^, such that decreased photosynthesis is correlated with increased disease severity and increased virus multiplication^36,37^. JLCuGV infiltrated WT showed severe symptoms and significantly decreased photosynthesis as compared to amiRNA transgenics (C1/C4 and C2/C3). The V1/V2 transgenics showed mild symptoms and relatively less reduction of photosynthesis parameters as compared to WT. The decrease in photosynthesis rate has been previously reported in geminivirus-infected leaves of *Eupatorium makinoi*^35^ and mustard after turnip mosaic virus infection^37^. Naidu et al*.*^38^ suggested that a reduced level of chlorophyll-a may be responsible for the reduced rate of photosynthesis.

The transpiration rate, WUE and stomatal conductance were significantly higher in virus-infected transgenics compared to WT. The reduced transpiration rate and stomatal conductance were also observed in mustard after geminivirus infection^37^. The Ci and Ci/Ca were significantly higher in WT, suggesting that the CO_2_ taken by the plants was not utilized during photosynthesis and got accumulated in the cells, increasing the Ci and Ci/Ca ratio. It also correlates with the reduced rate of photosynthesis observed in WT. Similar results were reported with tobamovirus infection for reduced stomatal conductance, transpiration rate and Ci in pepper^39^. The ɸCO_2_ was significantly higher in transgenics, suggesting efficient CO_2_ assimilation, which correlated with both increased photosynthesis rate and reduced Ci of transgenics. The reduced ɸCO_2_ is reported for pea on *Pea enation mosaic virus* (family *Luteoviridae*) infection^40^. The higher ETR seen in amiRNA transgenics suggests better redox homeostasis. The high ETR was found associated with improved reactive oxygen species (ROS) homeostasis during salinity^41^. Although Guo et al*.*^37^ report no change for ETR during geminivirus infection, a significant reduction in ETR was observed in grapevine on virus infection^42^.

Fv/Fm was maintained at a healthy 0.8 level^31^ in both WT and transgenics even after virus infiltration. Similarly, no change in Fv/Fm was reported for geminivirus infected mustard^37^, and *Pepper mild mottle virus* (PMMoV) infected pepper^39^. ɸPSII was significantly higher in transgenics, suggesting proper functioning of PSII as it is a measure of PSII performance. In WT, ɸPSII was significantly reduced, and reduced ɸPSII is reported to cause the accumulation of reduced bound quinone, which causes damage to the primary electron acceptor plastoquinone of PSII^43^. Therefore, reduced ɸPSII can be correlated to the reduction of ETR observed in WT during infection. The qP indicates the efficient conversion of light into chemical energy^37^ and significantly improved qP in transgenics can be attributed to the maintenance of photochemical quenching followed by increased photosynthesis rate in transgenics. The overexcitation of the photochemical system after turnip mosaic virus infection caused reduced qP in mustard^37^.

In WT, the sugar content was significantly increased and intermediates of the tricarboxylic acid (TCA) cycle (malic acid, pyruvic acid and citric acid) reduced after virus infiltration. This suggests that sugar metabolism is affected and the normal functioning of the TCA cycle is disturbed in WT after JLCuGV infection. In transgenics, no remarkable changes are observed, indicating better maintenance of metabolic pathways on virus infiltration. The increase in sugar content of WT is correlated with the virus infection in *Tobacco mosaic virus*-infected tobacco^44^, *Tobacco rattle virus*-infected Arabidopsis^45^ and CMV infected beet plants^46^. Similarly, the decrease in carboxylic acids of WT is correlated with virus infection in *Papaver somniferum* on geminivirus *Ageratum enation virus* (AEV) infection^47^. The decrease in sucrose concentration is reported in *Potato virus Y* (PVY)-inoculated potato leaves, while the increase in sucrose content reported in Jatropha infected by geminivirus^48^. In WT, a decrease in sucrose and an increase in fructose and glucose content after virus infiltration might be due to activation of invertase enzyme which converts sucrose into fructose and glucose^49,50^. An increase in glucose concentration is also reported in tomato infected with *Tomato mosaic virus*^50^ and PVY-inoculated potato leaves^51^.

In conclusion, the study reports that a new isolate of JLCuGV was responsible for the leaf curl disease in Jatropha CP9 accession. All six ORFs of JLCuGV genomic DNA exhibited RSS activity *in-planta*. Three amiRNAs were designed against C1/C4, C2/C3 and V1/V2 transcripts by targeting the overlapping regions. Transgenics expressing these amiRNAs showed a resistance (C1/C4 and C2/C3) and tolerance (V1/V2) response to viral infection. A direct correlation of the viral load and disease symptoms with relative levels of amiRNA was observed. The photosynthetic parameters were significantly better in amiRNA transgenics as compared to WT on virus infiltration, suggesting that both light and dark centres of photosynthesis are maintained due to reduced viral load. Likewise, the metabolite profiles were not much altered in amiRNA transgenics, while sugar metabolism is disturbed in WT on virus infiltration, again suggesting resistance of the amiRNA transgenics to the virus. MSAP analysis revealed an increase in methylation and demethylation profiling in amiRNA transgenics as compared to VA and WT, suggesting that methylation pathways get activated in amiRNA transgenics, and increased level of methylation profiling is correlated with a degree of virus resistance in amiRNA transgenics. All the results indicated that the amiRNA transgenics were better adapted to resist/tolerate JLCuGV infection. Importantly, as amiRNAs designed in this study share homology to related geminiviruses that infect other crops like tomato, pepper and papaya, so these have the potential to be employed for developing geminiviral resistance in other susceptible economically important crops.

## Materials and methods

### Plant material

The *J. curcas* leaves CP9 accession showing mosaic and leaf curl symptoms were collected from Nesvad, Jatropha experimental fields of Plant Omics division, CSIR-CSMCRI, Gujarat. Collections were made during July and September 2014. The collected samples were stored at − 80 °C until use.

### Detection and identification of begomoviruses

The genomic DNA of the collected samples was isolated by modified CTAB method^51^. The detection and characterization of begomoviral DNA components (DNA-A, DNA-B, DNA- β) from the samples was performed by PCR with degenerate primers (Supplementary Table S3) using 100 ng of genomic DNA. The complete genome of the begomovirus was amplified from the genomic DNA (100 ng) by RCA using a TempliPhi Amplification Kit (GE Life Sciences, UK). The RCA products were digested with *Hind*III to get the complete begomoviral genome (2.7 kb), cloned in pBluescriptII KS(+) vector and sequenced.

### Cloning of JLCuGV ORFs independently in pCAMBIA-1301

The individual ORFs of JLCuGV genome (AV1, AV2, AC1, AC2, AC3 and AC4) were cloned in pRT101, thereafter, the entire cassette with CaMV35S promoter-ORF- PolyA was cloned in pCAMBIA-1301 vector using *Pst*I (AV1, AV2 and AC1) or *Hind*III (AC2, AC3 and AC4; Fig. 1a). The cloned ORF constructs were then mobilized in *A. tumefaciens* LBA4404 strain for agroinfiltration.

### Agroinfiltration of JLCuGV ORF constructs

The *Agrobacterium* cultures (OD_600_ = 1; 1 ml per plant) of individual JLCuGV ORF constructs were injected into leaves of stable *N. tabacum cv. Xanthi* GFP silenced lines into separate patches in each leaf for identification of RSS activity. Agroinfiltration was performed by a method described by Hamilton et al*.*^52^. The *Agrobacterium* cultures (OD_600_ = 1; 1 ml per plant) were injected into leaves of the *N. benthamiana* (WT and transgenics) at 4–6 leaf stage using a 2 ml syringe. The plants were kept in an insect-free chamber at a constant temperature (25–28 °C) under 14–16 h lighting.

### Detection and confirmation of RSS activity

The agroinfiltrated leaves of the GFP silenced lines were observed under UV light for visualization of green fluorescence at 7, 11 and 15 dpi. The ORFs having inherent RSS activity were expected to restore the GFP expression by suppressing the RNA silencing machinery.

Total RNA was isolated from the individual regions of leaves^52^ agroinfiltrated independently with single JLCuGV ORF (AV1, AV2, AC1, AC2, AC3 and AC4) and FHVB2 construct, a well-known RSS used as control. The cDNA was prepared using 5 μg of total RNA using SuperScript IV First-Strand synthesis system (Invitrogen), as per manufacturer’s instructions. cDNA (1 μl), from each sample was used for amplification of *gfp* gene (~ 300 bp) with specific primers (Supplementary Table S3). The cDNA from mock (only buffer infiltrated) leaves was used as a negative control as it lacked the GFP expression. The samples showing amplification of the *gfp* transcript confirmed the restoration of GFP expression in the agroinfiltrated region of the leaf, thereby confirming the inherent RSS activity of the particular ORF.

### In silico designing and construction of amiRNA plasmids for tobacco transformation

The common overlapping sequences between the two genes (AV1–AV2, AC1–AC4 and AC2–AC3) were used for designing amiRNA (Table 1, Supplementary Fig. S5a). The amiRNA design was carried out by using the WMD3 (Web MicroRNA Designer 3) online tool (http://wmd3.weigelworld.org). While designing of amiRNA, we also tried to take the sequences, which can be used to target the begomovirus infecting tomato, papaya, pepper, ageratum etc. (Supplementary Fig. S5b–d), so that same amiRNA construct can also be used against other begomoviruses.

The three designed amiRNAs (Table 1) were amplified by four sets of primers (Supplementary Table S3) via overlapping PCR on the pNW55 vector backbone for obtaining pre-miRNA as given in Sharma et al*.*^53^. The pre-amiRNA fragment was then cloned in pRT101, using *Xho*I and *BamH*I sites for directional cloning, under CaMV-35S promoter. Then the entire cassettes of C1/C4, C2/C3 and V1/V2 amiRNA with CaMV-35S promoter were cloned in pCAMBIA-1301 using *Hind*III site. The schematic representation of the amiRNA gene construct in pCAMBIA-1301 showed in Supplementary Fig. S2.

### Tobacco transformation

Three amiRNA constructs (C1/C4, C2/C3 and V1/V2 amiRNA) cloned in pCAMBIA-1301 vector and vector backbone alone (VA) were mobilized into the *A. tumefaciens* strain LBA4404. The transformed agrobacterium was used for the transformation of *N. benthamiana* leaf discs, according to Clemente^54^.

### Molecular validation of transgenic plants expressing the amiRNA

The transgenic plants (T_1_) were checked by GUS assay to confirm the transgene expression. These were later confirmed for gene integration by PCR. The PCR was carried out using the primers specific for amiRNA gene, *gus gene and hygromycin* (*hptII*) gene (Supplementary Table S3). The positive lines were then checked for copy number by quantitative Real-Time PCR (qRT-PCR).

### Construction of infectious clone for agroinfiltration

The construction of infectious clone was performed in two steps. In the first step, the RCA product was double digested with *BamH*I and *Hind*III, then the digested product (0.65 kb) was cloned in pCAMBIA-1301 to get a recombinant vector pCAM-JLCuGV-Partial. In the second step, the full-length genome of RCA product generated by *Hind*III was ligated with pCAM-JLCuGV-Partial, to get a complete infectious clone pCAM-JLCuGV-Inf. The orientation of the inserts was confirmed with sequencing. The positive clones that contain tandemly repeated viral genomes having two *ori* (origin of replication) sites were selected. The infectious clone was transformed in *A. tumefaciens* LBA4404 strain. For causing infection, the agroinfectious clone of JLCuGV (Supplementary Fig. S4) was infiltrated in leaves of WT and transgenic lines of each amiRNA (L61, L64, L67 of C1/C4 amiRNA; L41, L42, L44 of C2/C3 amiRNA and L13, L14, L15 of V1/V2 amiRNA) in three replicates.

### Molecular confirmation of JLCuGV replicating in agroinfiltrated tobacco plants

The genomic DNA was isolated from the tobacco plants^55^ agroinfiltrated with JLCuGV infectious clone. The presence of an infectious clone of JLCuGV was checked by PCR with specific degenerate primers (Supplementary Table S3) using 100 ng of each genomic DNA as a template. The *actin* gene was amplified as a loading control using NtActF and NtActR primers (Supplementary Table S3). The complete begomoviral genome was amplified by RCA using a TempliPhi Amplification Kit (GE Life Sciences, UK). The amplified RCA product was digested with *Hind*III to get 2.7 kb genomic DNA.

### Determination of viral load

Geminivirus copy number in the agroinfiltrated plant was determined as reported in Legarrea et al*.*^56^. The pCAMBIA-1301 containing JLCuGV genomic DNA plasmid was linearized with *Bgl*II restriction enzyme. The quantified plasmid DNA was used to calculate the number of copies based on the formula:

$${\text{Number of copies}} = \frac{({\text{DNA (ng)}} \times 6.022 \times 10^{{23}})}{({\text{length of a vector (bp)}} \times 109 \times 650)},$$

where 650 denotes weight (Da) of a base pair.

The standard curve was prepared by serial dilution of 10^9^ to 10^1^ plasmid of pCAMBIA-1301 containing JLCuGV genomic DNA. The qRT-PCR reaction performed using the AV2 gene primers (Supplementary Table S3). The copy number was quantified using the Ct (cycle threshold) value plotted against the standard curve formula. Three lines of each transgenic event were used for the analysis.

### Small RNA isolation

The total RNA was isolated from transgenic lines by Tris-SDS buffer^57^ and then the small RNA was isolated by using different concentrations of polyethylene glycol (PEG) according to Singh and Jha^58^. The quality of the purified RNA was examined by 1% agarose gel electrophoresis and the absorbance ratio (A_260_/A_280_ and A_260_/A_230_) using Epoch Microplate Spectrophotometer (BioTek, USA).

### The quantitative estimation of amiRNA

The cDNA was synthesized using small RNA as template and specifically designed stem-loop primers (Supplementary Table S3) by SuperScript IV First-Strand Synthesis System (Invitrogen). The quantitative estimation of amiRNA was carried out using cDNA with stem-loop primers based qRT-PCR as given in Czimmerer et al.^59^ and *actin* gene was used as an internal control.

### Gas exchange and chlorophyll fluorescence measurement

Photosynthetic gas exchange parameters were measured at photosynthetic photon flux density (PPFD) of 1000 μmol m^−2^ s^−1^ by the open infrared gas analyzer (IRGA, Model LI-6400XT, LI-COR). All other conditions for gas exchange and chlorophyll fluorescence measurement were kept the same as reported in Shukla et al*.*^60^. Photosynthetic gas exchange parameters and chlorophyll fluorescence parameters were recorded in WT and amiRNA transgenics (L61, L64, L67 of C1/C4, L41, L42, L44 of C2/C3 amiRNA and L13, L14, L15 of V1/V2) at 21, 28 and 35 dpi.

### Gas chromatography–mass spectrometry (GC–MS) analysis

The sample preparation and derivatization by N-methyl-N-trimethylsilyl trifluoroacetamide (MSFTA) was carried out as given in Lisec et al*.*^61^. The derivatized samples were then transferred into glass vials suitable for GC–MS analysis. The GC–MS analysis was carried out in a SHIMADZU prominent instrument (SHIMADZU Corporation, Kyoto, Japan) equipped with EI mass spectra and autosampler. The injection volume for each sample was 1.0 μl throughout the analysis. Gas chromatographic separation was performed using the silica capillary SH-Rtx-5 column (30 m × 0.25 mm)^62^. Injection temperature was 230 °C, the interface was set to 250 °C and the ion source was adjusted to 200 °C. Helium flow was 1 mL min^−1^. After a 5-min solvent delay time at 70 °C, the oven temperature was increased at 5 °C min^−1^ to 310 °C, 1 min isocratic, cool-down to 70 °C, followed by an additional 5-min delay. The ion source temperature was 200 °C and mass ions in the range of 45–600 m/z were scanned at a rate of 1.0 scans s^−1^.

### MSAP analysis

MSAP analysis was performed as reported earlier^25^. For MSAP analysis, WT, VA and two representative lines of C1/C4 (L61, L67), V1/V2 (L13, L15) and C2/C3 (L42, L43) amiRNA transgenics were selected. The DNA was isolated from each plant with and without virus agroinfiltration after 35 dpi.

### Statistical analysis

The mean values and standard deviations were calculated of three independent biological replicates and the bars in figures represent the mean ± SD. Analysis of variance was calculated using the Tukey test by InfoStat software (version 2018) at *P* ≤ 0.05 to determine the significance of the difference in viral load of WT, VA and V1/V2 amiRNA transgenics and relative expression of amiRNA in the C1/C4, C2/C3 and V1/V2 amiRNA transgenics at 14, 21, 28 and 35 dpi. Similarly, the analysis of variance was also calculated in the photosynthesis parameters between the means of control and virus infiltrated plants (WT, C1/C4, C2/C3 and V1/V2 amiRNA transgenics) at 21, 28 and 35 dpi. Different alphabets indicated mean values of treatments that were significantly different from each other.

## Supplementary information

Supplementary Information.
