# Supplementary material for: Artificial miRNA mediated resistance in tobacco against Jatropha leaf curl Gujarat virus by targeting RNA silencing suppressors
Source: Sci Rep. 2021 Jan 13;11:890. doi: 10.1038/s41598-020-79134-z (PMC7806619; doi:10.1038/s41598-020-79134-z)
Supplement: Supplementary file 1 — Supplementary Information. [file 41598_2020_79134_MOESM1_ESM.pdf]

Artificial miRNA mediated resistance in tobacco against *Jatropha leaf curl Gujarat virus* by targeting RNA silencing suppressors

Prashant More[a,b], Parinita Agarwal[a]*, Abhishek Anand[c], Neeti Sanan-Mishra[c] and Pradeep K Agarwal[ab]*

[a]Plant Omics Division,

CSIR-Central Salt and Marine Chemicals Research Institute (CSIR-CSMCRI),

Council of Scientific & Industrial Research (CSIR),

Gijubhai Badheka Marg,

Bhavnagar- 364 002, (Gujarat), INDIA

[b]Academy of Scientific and Innovative Research (AcSIR), Ghaziabad- 201002, India.

[c]Plant RNAi Biology Group,

International Centre for Genetic Engineering and Biotechnology

Aruna Asaf Ali Marg

New Delhi-110 067, India

***Corresponding author**

E-mail: pagarwal@csmcri.res.inn

E-mail: parinitaa@csmcri.res.in

Telephone +91-278-2564761,

Fax +91-278-2567562

Supplementary Tables

Table S1. Details of the MSAP primers used for methylation polymorphism analysis

**MSAP Primer/ Adaptor**	**Sequence of the primer (5'-3')**
Eco-Adaptor1	GACGATGAGTCTAGAA
Eco- Adaptor2	CGTTCTAGACTCATC
HM- Adaptor1	CTCGTAGACTGCGTACC
HM- Adaptor2	AATTGGTACGCAGTC
Preamp-F	GACTGCGTACCAATTCA
Preamp-R	GATGAGTCTAGAACGGT
MSAP-1	P1: GACTGCGTACCAATTCACC<br>P2: GATGAGTCTAGAACGGTCT
MSAP-2	P1: GACTGCGTACCAATTCAGA<br>P2: GATGAGTCTAGAACGGTCA
MSAP-3	P1: GACTGCGTACCAATTCAGC<br>P2: GATGAGTCTAGAACGGTGA
MSAP-4	P1: GACTGCGTACCAATTCACC<br>P2: GATGAGTCTAGAACGGTGA
MSAP-5	P1: GACTGCGTACCAATTCAGA<br>P2: GATGAGTCTAGAACGGTCT
MSAP-6	P1: GACTGCGTACCAATTCACT<br>P2: GATGAGTCTAGAACGGTAT
MSAP-7	P1: GACTGCGTACCAATTCACG<br>P2: GATGAGTCTAGAACGGTAA
MSAP-8	P1: GACTGCGTACCAATTCAGT<br>P2: GATGAGTCTAGAACGGTAG
MSAP-9	P1: GACTGCGTACCAATTCACT<br>P2: GATGAGTCTAGAACGGTAG
MSAP-10	P1: GACTGCGTACCAATTCACG<br>P2: GATGAGTCTAGAACGGTAT
MSAP-11	P1: GACTGCGTACCAATTCAAT

	P2: GATGAGTCTAGAACGGTTC
MSAP-12	P1: GACTGCGTACCAATTCAGT<br>P2: GATGAGTCTAGAACGGTAA
MSAP-13	P1: GACTGCGTACCAATTCACC<br>P2: GATGAGTCTAGAACGGTCA
MSAP-14	P1: GACTGCGTACCAATTCAGA<br>P2: GATGAGTCTAGAACGGTGA
MSAP-15	P1: GACTGCGTACCAATTCAAG<br>P2: GATGAGTCTAGAACGGTCT

Table S2. The concentration of all the detected metabolites in healthy (control) and virus infiltrated leaf tissue of WT and amiRNA transgenics

Sr. No.	Class of compound	Compound name	Concentration (µg/gm of tissue)							
			Control				Virus infiltrated			
			WT	V1	C1	C2	WT	V1	C1	C2
1	Sugars	Sucrose	397.08	228.62	139.12	229.99	204.07	293.86	165.91	289.23
2		D-Fructose	51.69	56.65	12.71	72.10	453.04	166.09	7.86	81.37
3		d-Glucose	47.42	36.26	14.88	42.54	383.98	87.67	9.85	44.93
1	Caboxylic acids	4-Aminobutanoic acid	2.70	0.97	0.29	6.13	2.61	1.70	3.94	3.26
2		Malic acid	119.09	137.84	264.65	219.26	68.09	88.06	204.29	195.28
3		Quininic acid	35.12	55.59	9.41	3.24	18.79	12.97	6.13	10.22
4		Malonic acid	0.80	1.15	0.29	0.88	0.56	0.36	0.41	0.59
5		pyruvic acid	9.55	4.59	2.53	2.72	0.54	1.55	1.44	1.33
6		alpha.-Ketoisovaleric acid	2.15	1.85	0.53	1.66	0.98	0.68	1.54	1.93
7		Citric acid	0.33	0.71	2.65	0.61	0.69	4.29	2.24	1.63
1	Polyols	Glycerol	72.70	82.76	70.29	67.18	66.34	21.50	18.14	14.07
2		2,3-Butanediol	0.55	1.15	0.76	0.53	0.98	0.61	0.63	0.89
3		Myo-Inositol	125.95	161.74	221.12	205.14	280.02	199.32	180.31	268.30
4		D-Mannitol	3.30	6.26	1.06	9.37	17.30	23.94	16.25	23.26
1	Fatty alcohols	11-Methyldodecanol	4.27	1.85	1.00	1.93	3.93	2.83	1.16	2.52
2		3-Dodecanol	1.06	1.06	3.06	0.88	4.91	1.91	2.27	1.63
3		3-Octanol	1.69	3.44	2.18	1.14	1.56	1.91	2.09	0.89
1	Amino acids	L-Proline	1.48	2.03	1.71	1.40	1.94	3.21	1.49	3.41

1	Alkene	1-Undecene	1.18	4.41	3.18	2.28	1.23	2.07	2.02	1.04
2		3-Eicosene	1.40	0.53	2.53	0.61	4.45	1.78	2.15	2.37
3		1-Nonene	1.18	0.79	0.94	1.49	1.93	1.91	1.49	1.63
1	Fatty acids	Stearic acid	11.57	14.74	9.60	10.60	0.00	0.00	0.00	0.00
2		Myristic acid	0.25	0.53	0.53	0.44	0.00	0.00	0.00	0.00

Table S3. List of primers used in the present study

Primer Name	Primer sequence (5'-3')	Purpose
AV 494<br>AC 1048	GCCYATRTAYAGRAAGCCMAG<br>GGRTTDGARGCATGHGTACATG	Detection of DNA-A
beta01<br>beta02	GGTACCACTACGCTACGCAGCAGCC<br>GGTACCTACCCTCCCAGGGGTACAC	Detection of DNA-β
PCRc1<br>PBL1v2040	CTAGCTGCAGCATATTTACRARWATGCCA<br>GCCTCTGCAGCARTGRTCKATCTTCATACA	Detection of DNA-B
V1/V2-I miR-s<br>V1/V2-II miR-a<br>V1/V2-III miR-s<br>V1/V2-IV miR-a	AGTCGAAGTTCAGACGGCGACGTCAGGAGATTCAGTTTGA<br>TGACGTCGCCGTCTGAACTTCGACTGCTGCTGCTACAGCC<br>CTACGTCCCCGACTGAACTTCGATTCCTGCTGCTAGGCTG<br>AATCGAAGTTCAGTCGGGGACGTAGAGAGGCAAAAGTGAA	Amplification of V1/V2 amiRNA
C2/C3-I miR-s<br>C2/C3-II miR-a<br>C2/C3-III miR-s<br>C2/C3-IV miR-a	AGTCTCCCAGGTATAGACGCCATCAGGAGATTCAGTTTGA<br>TGATGGCGTCTATACCTGGGAGACTGCTGCTGCTACAGCC<br>CTATGGCCTCTTTACCTGGGAGATTCCTGCTGCTAGGCTG<br>AATCTCCCAGGTAAAGAGGCCATAGAGAGGCAAAAGTGAA	Amplification of C2/C3 amiRNA
C1/C4-I miR-s<br>C1/C4-II miR-a<br>C1/C4-III miR-s<br>C1/C4-IV miR-a	AGGAGCTGGATTTAGCTCCCTGACAGGAGATTCAGTTTGA<br>TGTCAGGGAGCTAAATCCAGCTCCTGCTGCTGCTACAGCC<br>CTTCAGGCAGCAAAATCCAGCTCTTCCTGCTGCTAGGCTG<br>AAGAGCTGGATTTTGCTGCCTGAAGAGAGGCAAAAGTGAA	Amplification of C1/C4 amiRNA
G-4368<br>G-4369	CTGCAAGGCGATTAAGTTGGGTAAC<br>GCGGATAACAATTTCACACAGGAAACAG	Amplification of pre-amiRNA
G11491-Xho1<br>G11494-Kpn1	TCCTCGAGCAGCAGCAGCCACAGCAAA<br>TCGGTACCGCTGCTGATGCTGATGCCAT	Amplification of amiRNA gene
hptII F<br>hptII R	TTCTTTGCCCTCGGACGAGTG<br>ACAGCGTCTCCGACCTGATG	Amplification of hygromycin gene
GusA F<br>GusA R	GATCGCGAAAACTGTGGAAT<br>TGAGCGTCGCAGAACATTAC	Amplification of gus gene
NtActF<br>NtActR	GATTTGCTGGTGATGATGCTCC<br>GTCTCAAACATGATCTGTG TCATC	Amplification of tobacco actin gene
GFP-F<br>GFP-R	GACCATGGCAAGTAAAGGAG<br>TCCGAGCTCTTAGAGTTCGTCG	Amplification of gfp gene
V1V2StemloopRT<br>C2C3StemloopRT<br>C1C4StemloopRT	GTTGGCTCTGGTGCAGGGTCCGAGGTATTCGCACCAGAGCCAACACGTCG<br>GTTGGCTCTGGTGCAGGGTCCGAGGTATTCGCACCAGAGCCAACATGGCG<br>GTTGGCTCTGGTGCAGGGTCCGAGGTATTCGCACCAGAGCCAACTCAGGG	For cDNA synthesis of amiRNA
V1V2qRT-F<br>C2C3qRT-F<br>C1C4qRT-F<br>Universal-qRT-R	GTTGTCGAAGTTCAGACGG<br>GTGGTCTCCCAGGTATAGA<br>GTGGGAGCTGGATTTAGCT<br>GTGCAGGGTCCGAGGT	For qRT PCR for relative estimation of amiRNA

Supplementary Figures

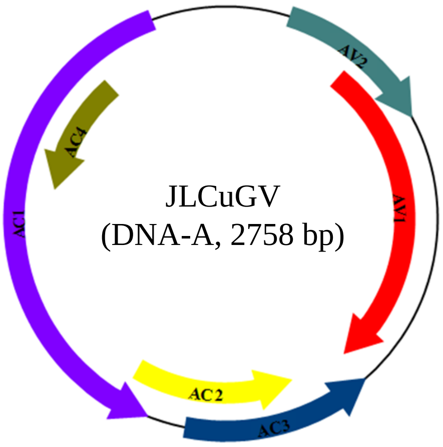

Figure S1. The genome organisation of JLCUGV DNA-A. The direction of arrow represents the orientation of ORF. This genome organisation map was constructed using Serial Cloner 2.6.1 software (freeware available at http://serialbasics.free.fr/Serial_Cloner.html).

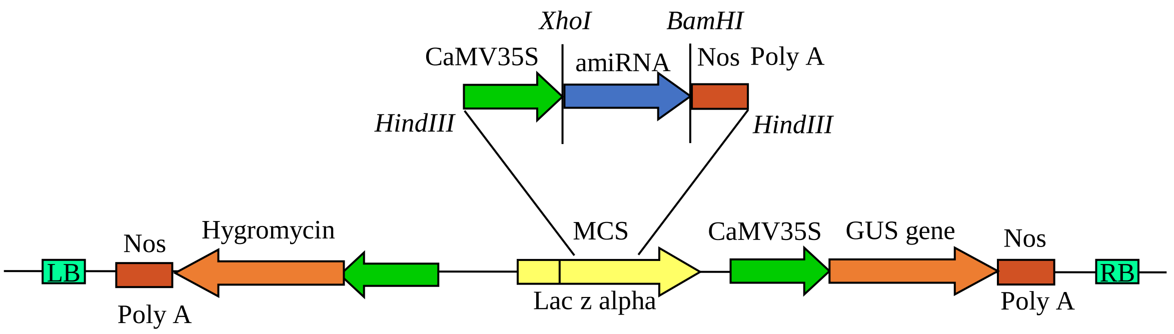

Figure S2. The schematic representation of amiRNA gene construct in pCAMBIA 1301.

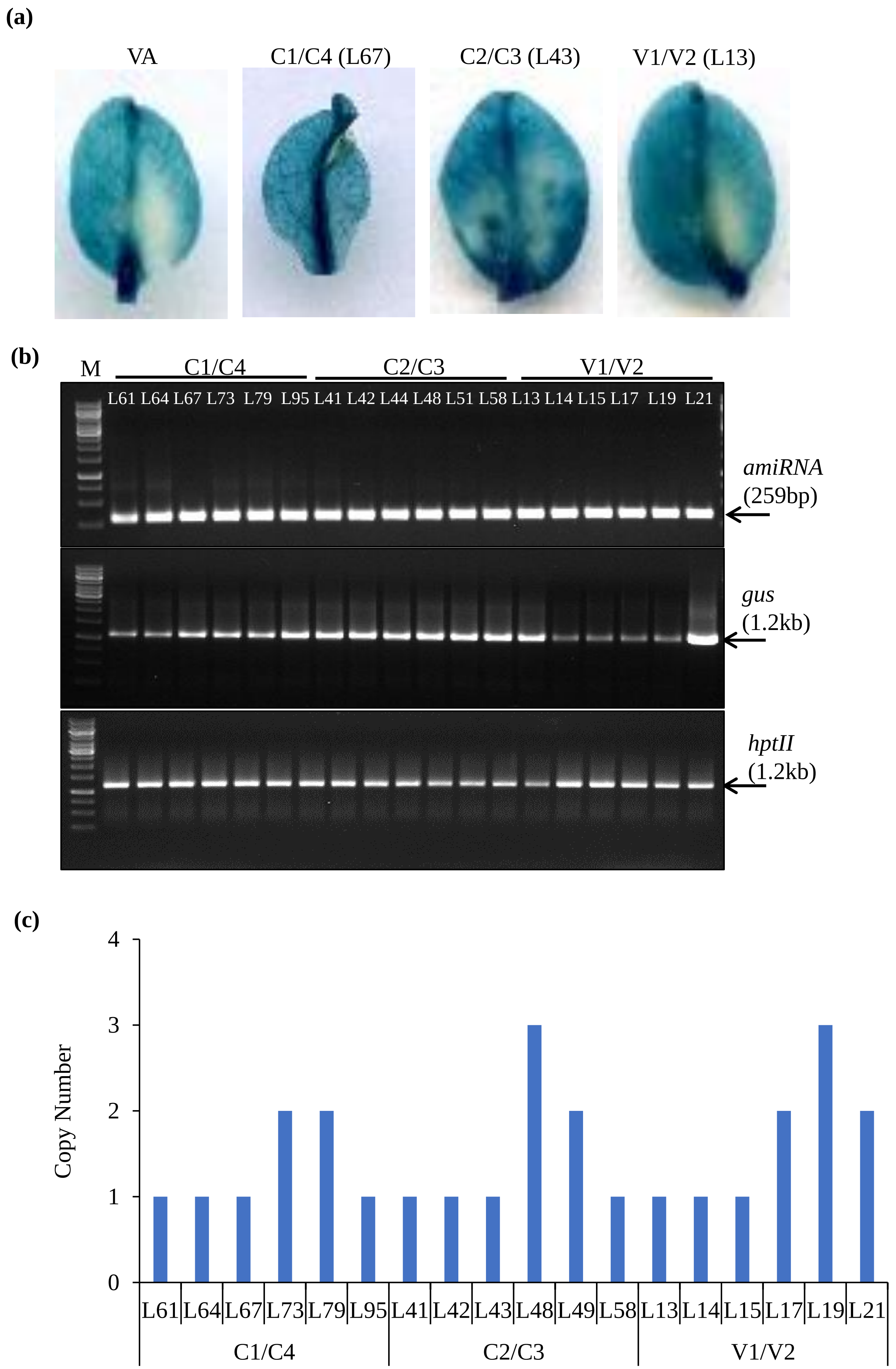

Figure S3. The confirmation of tobacco transgenics with amiRNA integration by GUS assay (a), amplification of integrated amiRNA gene, *gus* and *hptII* gene (b) and the copy number of transgenic lines of three transgenic events (c).

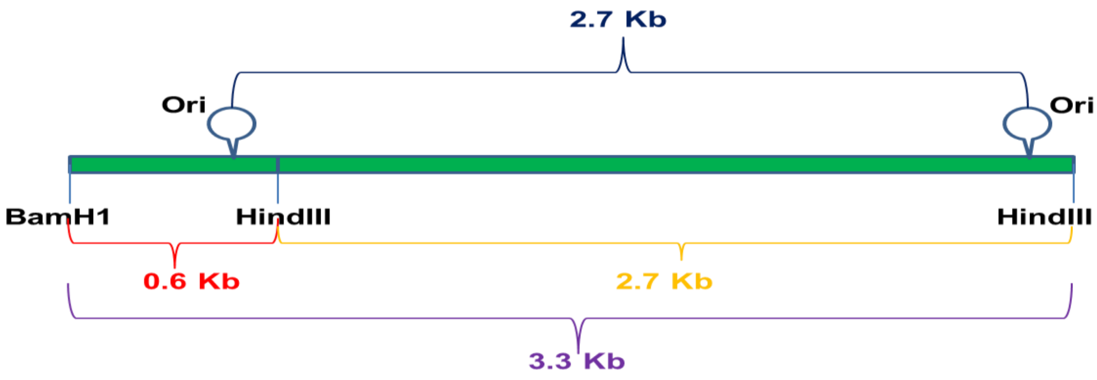

Figure S4. Schematic representation of infectious clone of DNA-A of JLCuGV. The hairpin structure represents the origin of replication (*ori)* of geminivirus.

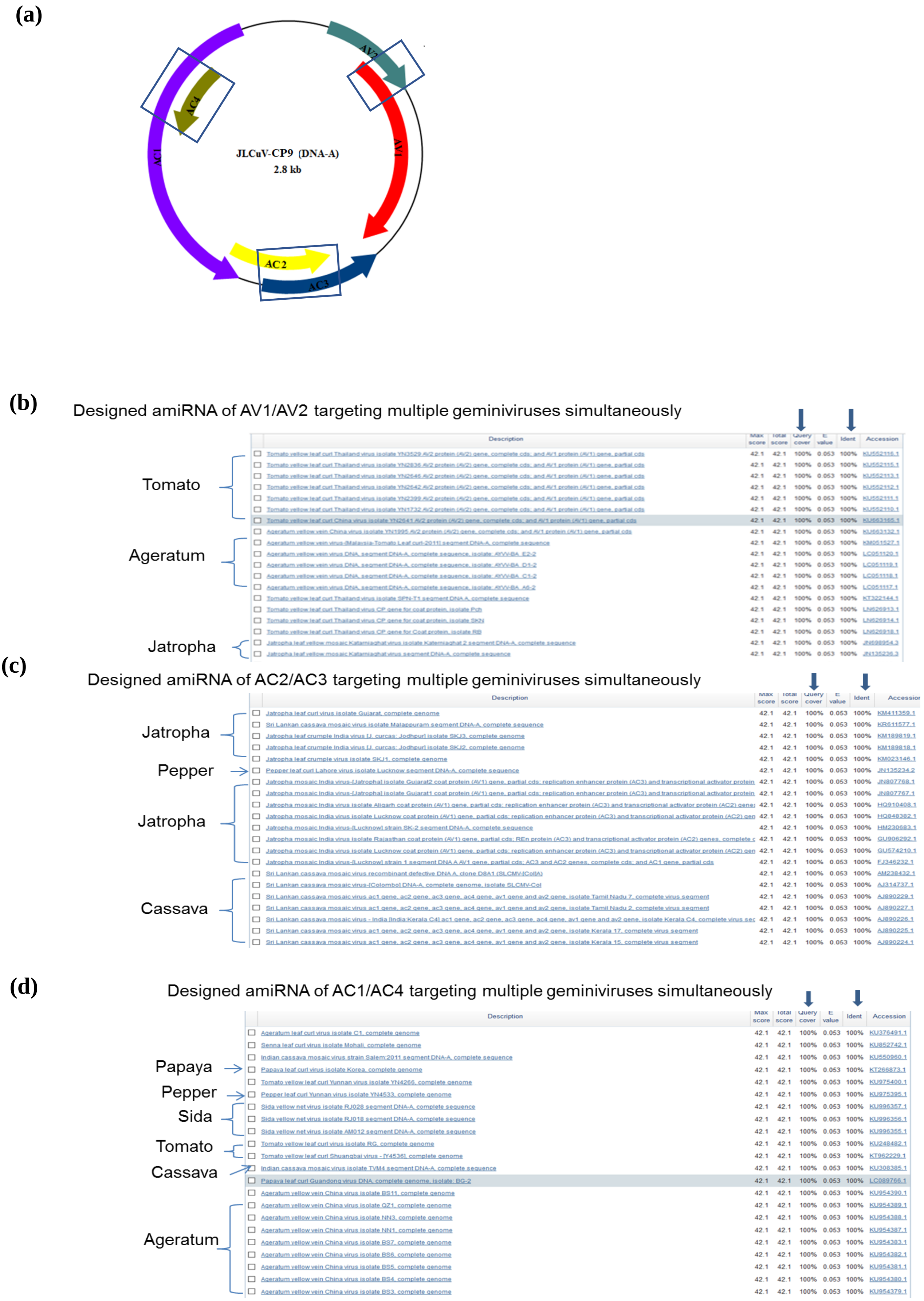

Figure S5. Designing and construction of artificial miRNA against the identified silencing suppressors by taking the overlapping regions used to generate three amiRNAs to target six genes (a), the three amiRNA designed as V1/V2 (b), C2/C3 (c) and C1/C4 (d), which can target the geminiviruses of other economically important crops like tomato, papaya, pepper, cassava. The genome organisation map (a) was constructed using Serial Cloner 2.6.1 software (freeware available at http://serialbasics.free.fr/Serial_Cloner.html).

Original Figures

GFP

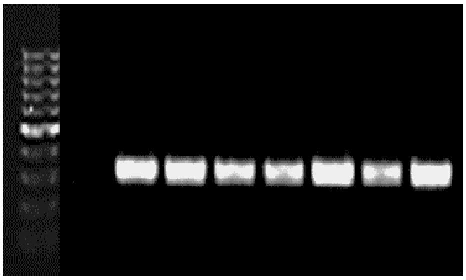

Actin

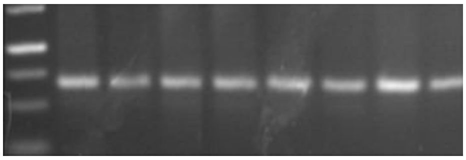

Original Fig. 1c: It is a part of Fig. 1c

PCR

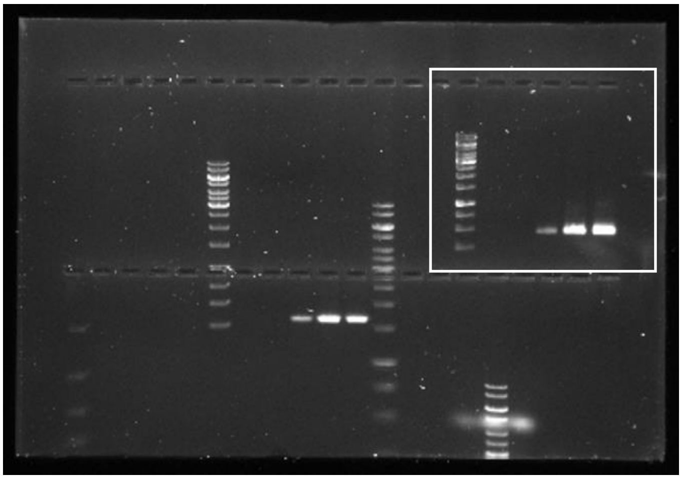

Actin

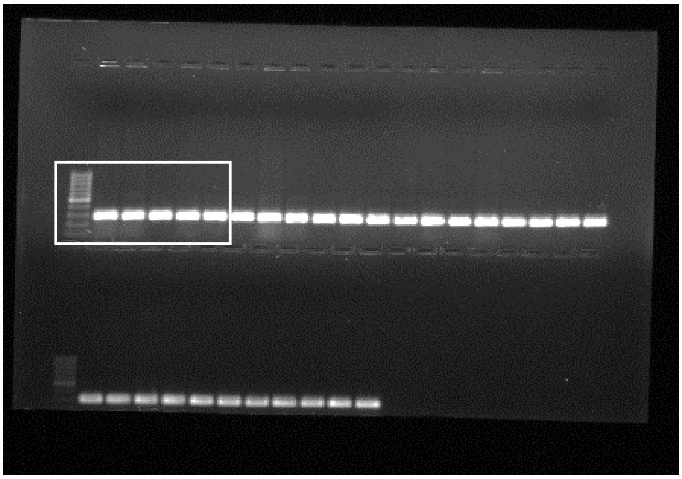

Original Fig. 2f: The boxed area is a part of Fig. 2f

RCA

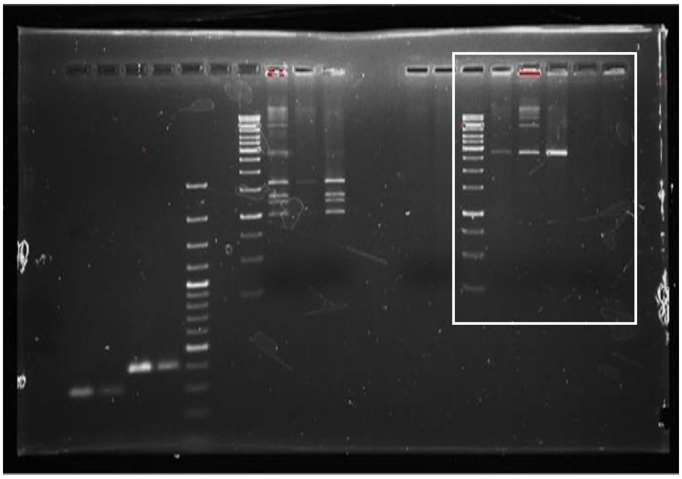

Actin

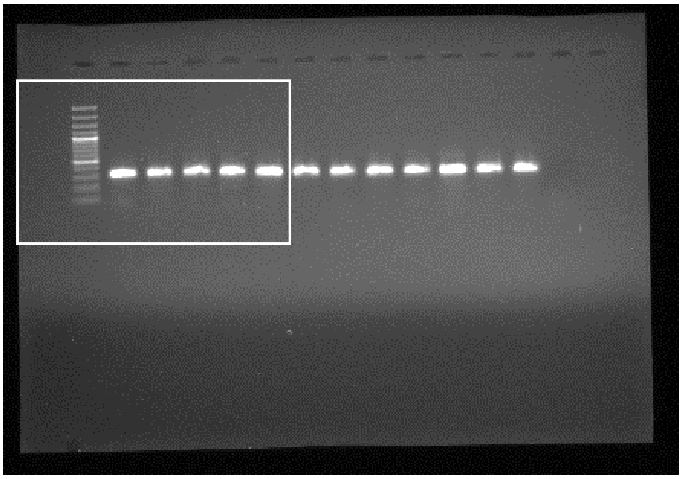

Original Fig. 2g: The boxed area is a part of Fig. 2g

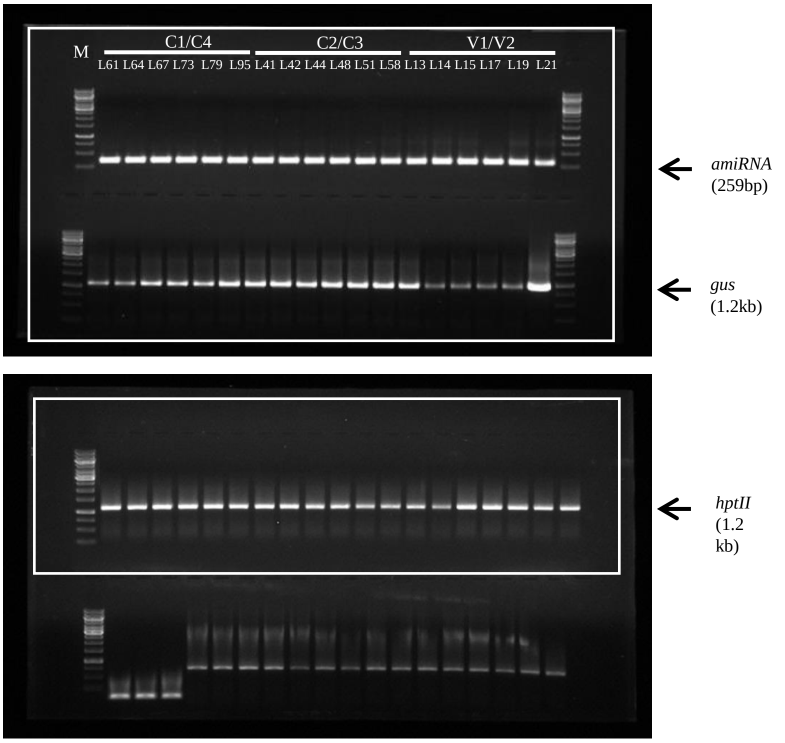

Original Fig. S3b: The boxed area is a part of Fig. S3b
